# Maxillomandibular Fixation Using Orthodontic Brackets as a Treatment Option for Mandibular Fractures

**DOI:** 10.7759/cureus.83085

**Published:** 2025-04-27

**Authors:** Kshitij Bang, Ramakrishna Shenoi, Alvina V Waghchoure, Nimish Situt

**Affiliations:** 1 Oral and Maxillofacial Surgery, Vidya Shikshan Prasarak Mandal (VSPM) Dental College and Research Centre, Nagpur, IND

**Keywords:** closed reduction, conservative management, mandibular fracture, maxillomandibular fixation, orthodontics brackets

## Abstract

Maxillomandibular fixation (MMF) is a treatment option for mandibular fractures. Various techniques can be used for MMF which include arch bars, eyelets, and intermaxillary fixation (IMF) screws. When a patient undergoing orthodontic treatment experiences a mandible fracture, conventional MMF wiring techniques cannot be employed without removal of the existing orthodontic brackets. In such cases, the existing orthodontic brackets can be utilized for MMF. To the best of the author’s knowledge, this is the first documented case in the literature where a mandible fracture in a patient undergoing orthodontic treatment was treated using the existing orthodontic brackets for MMF.

## Introduction

Fracture of the mandible is one of the most frequent types of jaw fractures, typically caused by falls, violence, and road traffic accidents. This type of fracture can be managed conservatively with maxillomandibular fixation (MMF) or surgically with open reduction and internal fixation (ORIF). Despite extensive research aimed at determining the best treatment method, the issue still remains controversial. Both treatment options can result in complications such as deviation of the chin, facial asymmetry, reduced movements of the jaw, temporomandibular joint (TMJ) dysfunction, ankylosis, chronic pain, and malocclusion [1].

While ORIF might appear to be superior to closed reduction (CR), it’s important to recognize that choosing ORIF significantly increases treatment costs. This is due to longer operating room time, more expensive surgical hardware, and prolonged general anesthesia. It also introduces a range of potential complications that must be carefully evaluated to determine whether the benefits of open surgery outweigh the surgical and postoperative risks. These complications can include injury to nerves and blood vessels, development of sialocele or salivary fistulas, facial scarring, hardware loosening, and infection [2].

Most surgeons appear to prefer managing condylar fractures without surgery. This tendency is mainly influenced by three key reasons. First, non-surgical approaches generally produce “satisfactory” outcomes in the majority of cases. Second, there is a lack of large-scale studies in the literature that track long-term outcomes following surgical intervention, likely because condylar fractures have traditionally been treated conservatively. Third, operating on condylar fractures is challenging due to the complex and risky anatomy of the area [3]. Conservative management is of utmost importance in the case of pediatric fractures. This modality of treatment is adopted in pediatric mandibular fractures to avoid postoperative functional or growth-related complications [4].

The conservative treatment includes performing MMF. There are various dental wiring techniques for achieving MMF, including Erich arch bars, eyelets, interdental wiring, acrylic splinting, orthodontic vacuum-formed thermoplastic splint, orthodontic brackets, and elastics [5]. Dental wiring techniques require passing wire around the tooth, which can cause periodontal injury and difficulties in maintaining oral hygiene. Improper adaptation can lead to orthodontic movement of the anterior teeth and mucosal ulceration from the wire ends. Additionally, there is a risk of percutaneous injury to the operator or assistant and a high chance of serological transmission of pathogens such as hepatitis B virus (HBV), hepatitis C virus (HCV), human immunodeficiency virus (HIV), and bacteria that can cause infection [6].

The placement of various dental wiring can be tedious for patients undergoing orthodontic treatment, as it often necessitates the removal of orthodontic brackets. Additionally, Erich arch bars can induce orthodontic movement of the teeth while applying forces for MMF [6], which poses a problem for patients with ongoing orthodontic treatment who also have a fracture of the mandible.

In this case report, we describe our experience in managing a case of mandible fracture of a patient undergoing orthodontic treatment. The authors have attempted to use the orthodontic brackets as anchorage devices for placement of the box wires required for MMF.

## Case presentation

**Figure 1:**
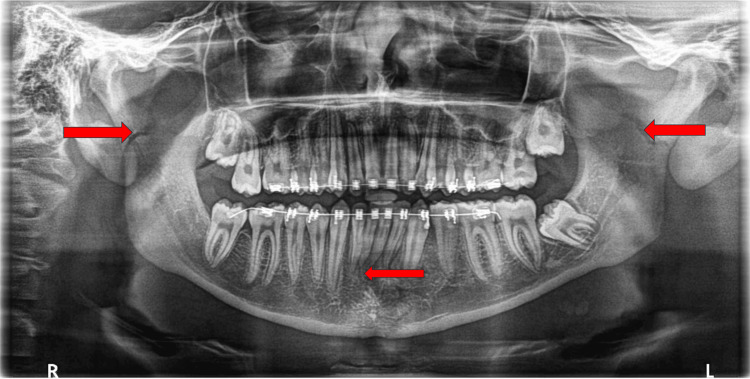
Orthopantomogram (OPG) showing a hairline fracture at the symphysis region of the mandible along with bilateral subcondylar fractures (red arrows).

A 19-year-old male patient undergoing orthodontic treatment reported a history of a road traffic accident due to a fall from a bike. On arrival at the outpatient department (OPD), the primary survey of trauma was done. The patient was conscious, oriented, and alert. The Glasgow Coma Scale (GCS) assessment of the patient was 15/15 (eye response 4, verbal response 5, motor response 6). Vital signs were normal. The patient reported no history of loss of consciousness, vomiting, convulsions, or seizures. No history of intra-oral bleeding was noted. A thorough medical history was taken, and no significant medical history was reported by the patient. Extra-orally, on inspection, the face was bilaterally symmetrical grossly. No evidence of any lacerations or abrasions was noted. Ocular movements, pupillary reflexes, and vision were normal. Lips were competent. Mouth opening was normal. On palpation, tenderness was noted in the symphysis region of the mandible as well as the condylar region bilaterally. No deviation in mouth opening was noted. On intra-oral examination, all teeth were present. No mobility was noted with any teeth. No intraoral contusions or lacerated wounds were noted. The occlusion was stable bilaterally, and the patient experienced pain while mastication or clenching. The mouth opening of the patient was restricted. On palpation, no step deformity or segmental mobility was noted with the maxilla and mandible. The patient was advised to undergo radiographic investigation, which further revealed a hairline fracture at the symphysis region of the mandible, along with subcondylar fractures bilaterally (Figure 1).

Considering certain factors, including that the fracture segments were undisplaced with a reproducible occlusion that returned to midline on release of the posterior force, CR and fixation were chosen for managing the case.

The placement of conventional arch bars, eyelets or intermaxillary fixation (IMF) screws necessitated the removal of the orthodontic brackets as these brackets would have caused a hindrance for the placement of the wires and the appliances. Hence, the previously placed orthodontic brackets were used for MMF. The placement of the wires for MMF was done by the following method.

Technique

**Figure 2:**
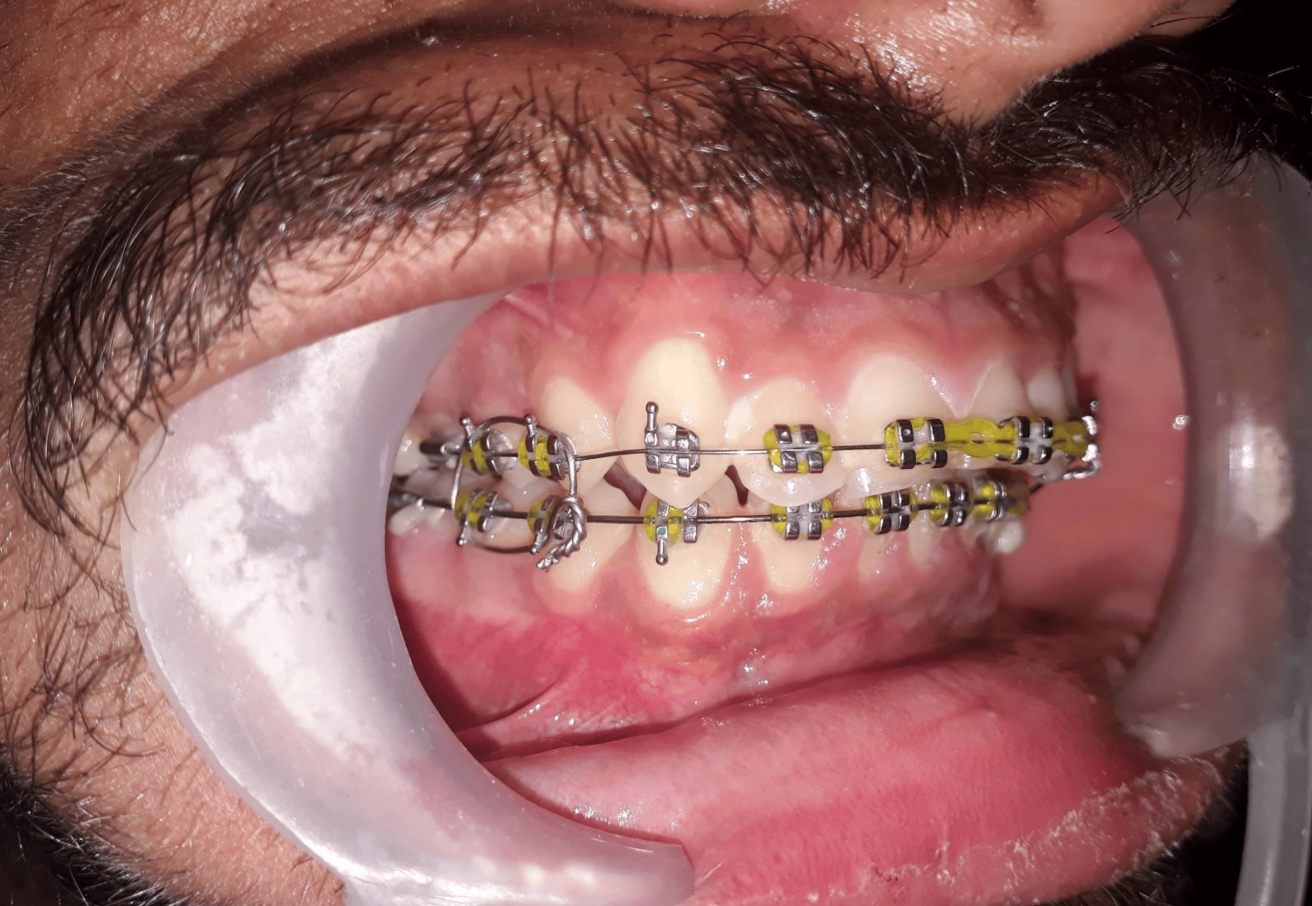
Orthodontic brackets used for maxillomandibular fixation (MMF) on the right side.

**Figure 3:**
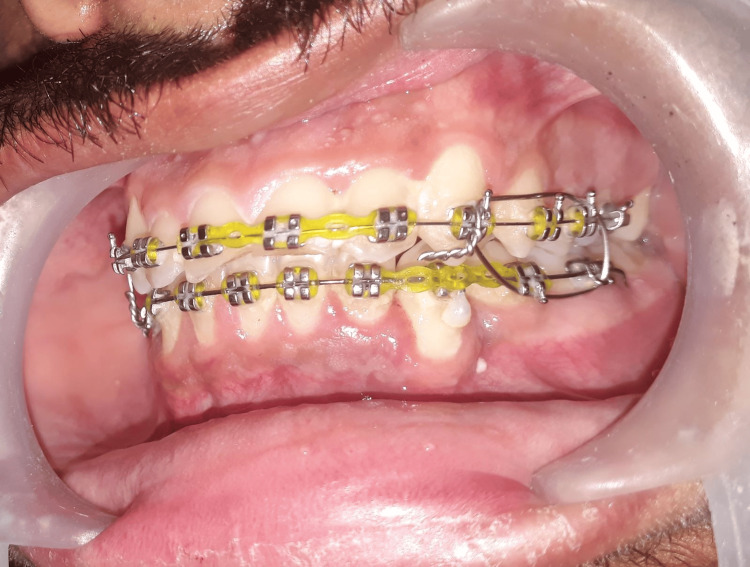
Orthodontic brackets used for maxillomandibular fixation (MMF) on the left side.

As the occlusion of the patient was stable and not deranged, the patient was asked to occlude the teeth, and the placement of the box wire required for MMF was done on either side by taking anchorage from the brackets. A minimum of four brackets were required on either side for achieving a stable MMF (Figures 2-3).

**Figure 4:**
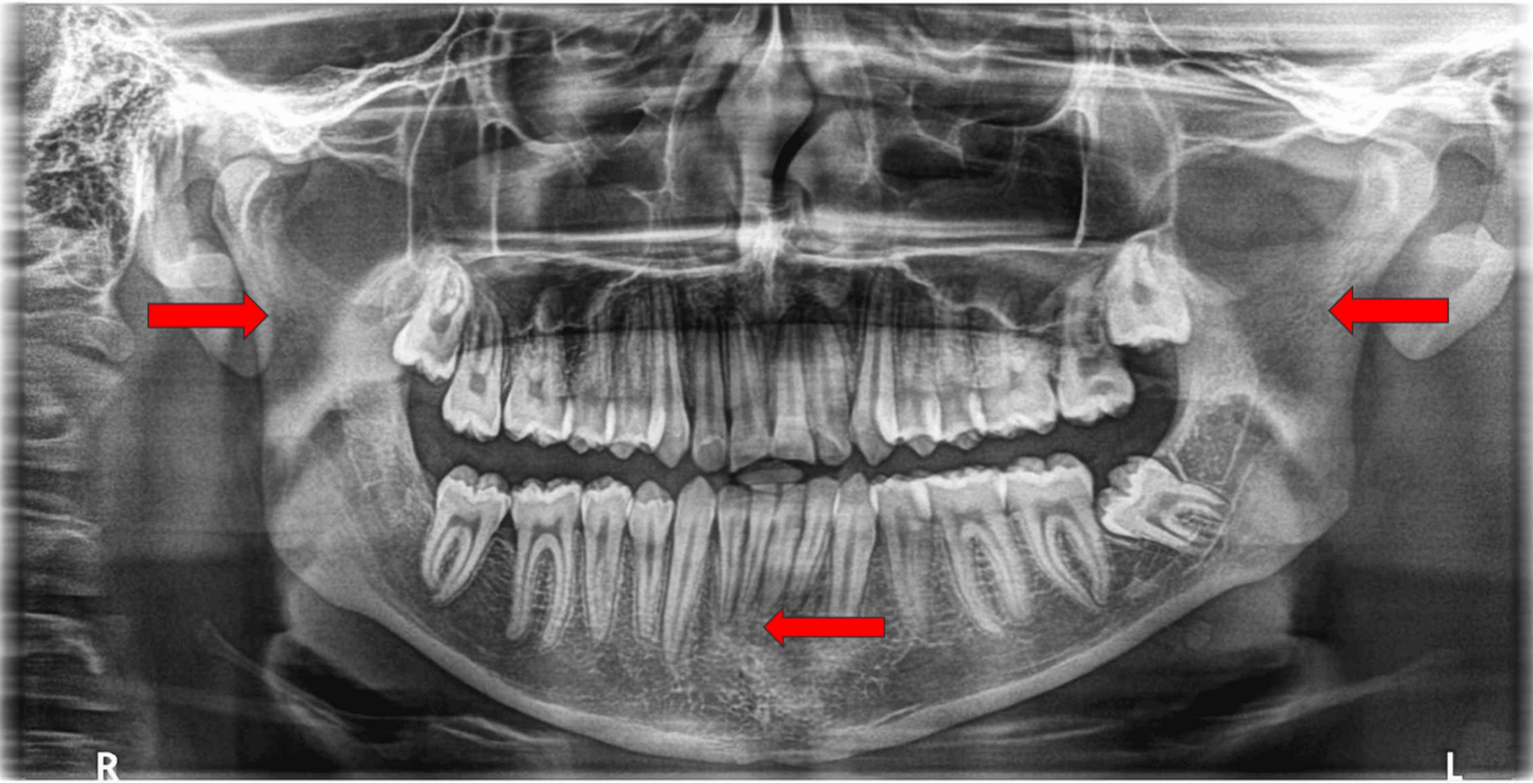
Nine-month postoperative orthopantomogram (OPG) showing bony union of the fractured mandible (red arrows).

The MMF was kept for a span of four weeks. The patient was advised a liquid diet orally with additional protein supplements to maintain daily caloric requirements. Instructions to maintain oral hygiene were given. Post the removal of the box wires of the MMF, the patient was kept on a soft diet for the initial two weeks post-release of MMF and was instructed to perform exercises for jaw mobilization. Radiographic investigations were carried out to assess the healing of the fractured bone postoperatively. The orthopantomogram revealed anatomic reduction at the fracture sites, and healing of the fractured segments of the bone was noted (Figure 4).

## Discussion

The primary dilemma in managing fractures, particularly in cases of mandibular condyle fractures, is deciding between open or CR and fixation. In the decision-making process, one typically weighs the risks against the benefits of each option [2]. ORIF is the preferred option owing to certain advantages like better anatomic reduction of the fractured segments, early return to work, better maintenance of oral hygiene, and overall superior functional clinical outcome [7].

However, any surgical procedure involving the mandibular condyle comes with a set of surgical and biological risks, along with additional disadvantages such as being more invasive, expensive, and possibly palsy of the facial nerve [7]. Surgical exposure of the TMJ is a complex task for a novice or unaided surgeon. Therefore, it requires the surgeon to aim for an acceptable outcome, which can be achieved through minimal surgical intervention [8]. In the present case, with undisplaced fracture segments and stable occlusion, the risks of managing the case with ORIF outweigh the benefits of it, hence, CR was chosen as a suitable treatment option.

The MMF can be performed with a variety of options like arch bars, eyelets, Leonard buttons, IMF screws, and splints [9,1]. MMF with orthodontic brackets can be considered as an additional treatment option for selected cases. It may be suitable for minimally displaced or favourable fractures where minimal reduction and stabilization forces are sufficient, as orthodontic brackets can withstand limited pulling forces [10]. Patient compliance is crucial and should be carefully assessed during case selection. Patients must adhere to given instructions, avoiding undue attempts to open their mouths, which could risk bracket breakage [4].

The use of existing orthodontic brackets for performing MMF allows for uninterrupted continuation of orthodontic treatment, reduces work and time required for conventional dental wiring, thus enhancing the comfort of the patient. Additionally, this approach minimizes the manipulation of wires, thereby lowering the risk of wire prick injuries, a common concern associated with handling wires [11,12]. In case scenarios, where the goal is to minimize the invasiveness, avoid prick injuries, and reduce treatment duration, such options should always be taken into account.

The cons of this technique include the limited strength of orthodontic brackets, which may not be suitable for managing complex or significantly displaced fractures. There is also a risk of bracket dislodgement or failure due to routine oral functions or patient noncompliance. Additionally, the technique may cause discomfort or inconvenience for the patient, particularly with dietary and hygiene restrictions.

The pros of this technique include uninterrupted orthodontic treatment, allowing ongoing progress without the need to remove appliances. It also enhances patient comfort by avoiding bulky and uncomfortable conventional wiring. It reduces clinical time and effort through a simplified fixation process and lowers the risk of prick-related injuries by minimizing wire manipulation. Also, it offers a minimally invasive approach, which is suitable for cases requiring reduced procedural complexity. This technique is cost-effective by utilizing existing orthodontic appliances, and it also improves patient compliance due to a less cumbersome and more tolerable procedure.

## Conclusions

The orthodontic brackets can serve as a useful anchorage for MMF in case of patients with a fracture of the mandible undergoing orthodontic treatment. The orthodontic brackets can withstand the required pulling forces in undisplaced fractures or fractures requiring minimal reduction of the bone. These brackets eliminate the need for placement of appliances and wires required for MMF, which in turn lowers the risk of prick injuries, aligning with the goals of minimizing invasiveness and treatment duration.
